# A Framework for Digital Health Policy: Insights from Virtual Primary Care Systems Across Five Nations

**DOI:** 10.1371/journal.pdig.0000382

**Published:** 2023-11-08

**Authors:** Divya Srivastava, Robin Van Kessel, Marine Delgrange, Avi Cherla, Harpreet Sood, Elias Mossialos

**Affiliations:** 1 Department of Health Policy, London School of Economics and Political Science (LSE), London, United Kingdom; 2 LSE Health, London School of Economics and Political Science (LSE), London, United Kingdom; 3 Department of International Health, Care and Public Health Research Institute (CAPHRI), Maastricht University, Maastricht, Netherlands; 4 Guy’s and St Thomas’ NHS Foundation Trust and NHS Leadership Academy, London, United Kingdom; Iran University of Medical Sciences, IRAN (ISLAMIC REPUBLIC OF)

## Abstract

Digital health technologies used in primary care, referred to as, virtual primary care, allow patients to interact with primary healthcare professionals remotely though the current iteration of virtual primary care may also come with several unintended consequences, such as accessibility barriers and cream skimming. The World Health Organization (WHO) has a well-established framework to understand the functional components of health systems. However, the existing building blocks framework does not sufficiently account for the disruptive and multi-modal impact of digital transformations. In this review, we aimed to develop the first iteration of this updated framework by reviewing the deployment of virtual primary care systems in five leading countries: Canada, Finland, Germany and Sweden and the United Kingdom (England). We found that all five countries have taken different approaches with the deployment of virtual primary care, yet seven common themes were highlighted across countries: (1) stated policy objectives, (2) regulation and governance, (3) financing and reimbursement, (4) delivery and integration, (5) workforce training and support, (6) IT systems and data sharing, and (7) the extent of patient involvement in the virtual primary care system. The conceptual framework that was derived from these findings offers a set of guiding principles that can facilitate the assessment of virtual primary care in health system settings.

## Author summary

Remote consultations in primary care feature in some health systems for several years. With the arrival of Covid-19, there was a concerted widespread effort to move towards remote consultations. But there may be negative impacts on patient care when consultations are not in person. To better understand the impact of remote consultations in primary care we conducted a narrative review and map the findings against the World Health Organization framework of the functional features of health systems. We develop the first iteration of this updated framework that accounts for the heterogenous nature of digital transformations and review the deployment of virtual primary care systems in five leading nations: Canada, Finland, Germany and Sweden and the United Kingdom (England). Seven common themes emerge: the stated policy objectives, regulation and governance, financing and reimbursement, delivery and integration, workforce training and support, IT systems and data sharing, and the extent of patient involvement in the virtual primary care system.

## Introduction

Digital health technologies represent a growing market share due in part to rapid advances in wireless technology and computing power as well as increasing interest in the application of artificial intelligence (AI) in health systems and service delivery, but also patient interest in having faster and easier access to medical care [1]. Digital health technologies used in primary care (also termed virtual primary care [VPC]) allow patients to interact with primary healthcare professionals remotely and through various modes of communication such as email, text, online chat, video or phone calls [2]. With the COVID-19 pandemic reducing or removing the possibility face-to-face appointments, VPC options such as online consultations have become an important element of primary care service delivery [3], which are also actively sought out by their populations [4,5]. Since the start of the COVID-19 pandemic, two-thirds of European Union healthcare providers reported an increase in the adoption of digital technologies to engage with and support patients within their organisation [6]. A survey from the US reported that 80% of patients would like virtual consultations to continue post-pandemic and in Canada, up to one third would like virtual care to be the first point of contact after the pandemic [7,8]. However, the current iteration of VPC may also come with unintended consequences, such as accessibility barriers among patients with lower levels of digital literacy or complex conditions, adverse selection (relatively healthy patients opting out of the public system for virtual care), or cream-skimming [9,10].

The World Health Organization (WHO) has a well-established framework to understand the functional components of health systems, comprised of six building blocks (i.e., leadership and governance; health care financing; health workforce; medical products and technologies; information and research; and service delivery) [11], which are linked to four key health system functions (i.e., delivering services, creating resources, financing, and stewardship [12]. However, the existing framework does not sufficiently account for the disruptive and multi-modal impact of digital transformations across the various building blocks. Therefore, there is a distinct need to reinterpret and update this framework in the context of digital transformations and develop a novel framework with accompanying guiding principles to support a strengthened VPC system.

In this article, we aimed to develop the first iteration of this updated framework by reviewing the deployment of VPC systems in five leading countries: Canada, Finland, Germany, Sweden, United Kingdom (UK) with a focus on England [9,13–16]. The five countries were selected based on the extent of integration of the VPC system with the health system and the study team’s areas of expertise. They also reflect a mix of models of delivery, including different levels of decentralisation, financing, implementation, and user uptake [17–22].

## Methods

We performed a narrative review to identify gaps in the existing WHO building blocks framework and develop the conceptual framework. Five databases (PubMed, CINAHL, EBSCO, Web of Science, Cochrane Review) and Google Scholar were searched. Articles were included if they comprised empirical research, systematic reviews and review articles and were published between 2011 to the first quarter of 2022. Grey literature was identified through Google (Scholar) searches, websites of national institutions, and institutions that conduct health policy analyses (Table 1). Two authors (DS and MD) undertook the primary literature review and cross-checked each other’s findings.

**Table 1:** Literature search of the narrative review.

Academic query	Key words
*First query*	• [insert country name] “• digital health communication technology” OR “VPC” OR “virtual consultation” OR “remote consultation” OR “primary care” OR “ambulatory care” OR “outpatient care” OR “general practice” OR telemedicine OR “telehealth” OR “telecommunication” OR “ehealth” OR “e-health” OR “telemonitoring” OR “clinical decision support” OR “remote monitoring” OR “health financing” OR “privatisation” OR “privatization” OR “outsourcing”
*Second query*	• [insert country name] AND • (“digital” OR “virtual” OR “telemedicine” OR “telehealth” OR “telecommunication” OR “ehealth” OR “e-health” OR “telemonitoring” OR “remote” OR “communication technology” OR “communication technologies” OR “clinical decision support”) AND • (“primary care” OR “ambulatory care” OR consultation OR care OR “general practice” or “outpatient care”)
*FINAL query*	• [insert country name] AND • “digital” OR “virtual” OR “telemedicine” OR “telehealth” OR “telecommunication” OR “ehealth” OR “e-health” OR “telemonitoring” OR “remote” OR “communication technology” OR “communication technologies” OR “clinical decision support”) AND • (“primary care” OR “ambulatory care” OR consultation OR care OR “general practice” or outpatient care) AND • (“health financing” OR privatisation OR privatization OR outsourcing)
Grey literature query	• UK: Health Foundation, King’s Fund, Wellcome Trust, Nuffield Trust, NICE • Sweden: Research Institute of Industrial Economics • Finland: University of Tampere • Germany: Robert Koch Institute • Canada: Canadian Institute for Health Information

Findings of the review were first clustered in seven overarching categories, which were derived from the original WHO building blocks framework: (1) stated policy objectives, (2) regulation and governance, (3) financing and reimbursement, (4) delivery and integration, (5) workforce training and support, (6) IT systems and data sharing, and (7) the extent of patient involvement in the VPC system.

## Results

The narrative review identified forty relevant articles [8–47] (Table 2). The search includes a mix of academic papers (18 articles), grey literature and government documents (twenty-two). Grey literature supplemented the academic research for the five countries in this review.

**Table 2:** Summary of narrative review.

	Canada	Finland	Germany	Sweden	UK	Other (e.g., comparative papers	Total
Articles	1	1	1	4	5	6	18
Grey literature	3	5	6	1	5	2	22
	4	6	7	5	10	8	40

### Overview of findings

With respect to objectives, the studied countries reflect a mix of federal and sub-national arrangements in health policy planning and delivery. All studied countries had developed national digital health strategies at the time of this study [17–22]. All country strategies, however, do not discuss implications for VPC that consists of both public and private providers and platforms. With regards to regulation, all the studied countries have policies to regulate VPC. But, where there are decentralised arrangements, for example guidelines on the use of platforms, are drafted at the regional level as seen in Canada. Financing and reimbursement models vary across the studied countries: England, Canada, and Germany having aligned reimbursement of VPC with usual face-to-face consultation tariffs in a form of reimbursement parity. The cost implications of VPC systems mean that further evidence is needed to better understand their financial impact such as cost savings, or cost increases. Most countries have a mix of public and private providers. Germany, England and Sweden have private providers used in the public health system. Undoubtedly, the pandemic accelerated the use of VPC systems. Yet, the virtual care systems followed markedly different timelines between the studied countries. A common theme in all countries is that underserved populations remain a challenge for VPC including the interaction with the social determinants of health to access VPC. A VPC strategy that includes patient involvement is necessary to better meet the needs of patients of all ages and their conditions to support coordination in their pathway of care. Patient data sharing is essential, especially in countries with parallel public and private virtual consultation systems in which virtual care is delivered by physicians who may not be the usual provider such as in Canada, England and Sweden. This leads to difficulties around the continuity and coordination of care between online and face to face consultations, for example between the provider offering the online consultation and the patient’s regular GP.

### Country findings

#### Canada

In Canada, with regards to policy *objectives*, most decision-making is devolved to the provincial level, which leads to varying requirements across the country [16]. This has led to regional-level strategies and plans. This has an impact on *regulation*, for example guidelines on the use of platforms, are drafted at the regional level as seen in Canada. *Financing and reimbursement* led to the introduction of billing codes during the COVID-19 pandemic for the coverage of private virtual consultations by the public healthcare system [23]. In a federated system, as seen in Canada, *delivery and integration* have embarked on different approaches taken at the provincial level. There have been elements of telemedicine since the 1970s (e.g., between primary care providers in rural/remote areas and specialist doctors). The emergence of private digital health actors helps to fill a gap left by the lack of patient to doctor virtual options in the public sector [24]. The review did not identify evidence from the literature on *workforce* training support in Canada. *Data sharing/IT systems* is a pressing issue, especially where public and private virtual consultation systems run in parallel in which virtual care is delivered by physicians who may not be the usual provider [24]. Patient feedback on VPC consultations was absent in most cases due to the use of phone calls rather than apps or video consultation platforms by traditional GPs. (S1 Table).

#### Finland

Finland is a highly decentralised administrative health system. Out of the five country studies, Finland, stands out having had strategies updated over the years with public investment in the VPC infrastructure with clear central support and *objectives*. This strong steer from the centre supports *regulation*, where it works with a variety of stakeholders to support the provision of remote consultations. *Financing and reimbursement* for remote consultations in place for physicians, physiotherapists, psychologists and nurses. Patients can pay out-of-pocket (OOP) for private remote consultations in primary care. Private health care physicians accounted for a small proportion of all remote consultations. With regards to *delivery and integration*, public providers are more widespread. Patient involvement including providing feedback on virtual consultations is well established with systematic routine collection in Finland. Similar to Canada, the *workforce* is a mix GPs and nurses in virtual primary services; for example, nurses had the highest remote contacts in public municipal health centres in Finland and private providers employ older GPs with experience. A forward planning approach fosters e-health competencies among health care professionals with respect to training and ongoing support through knowledge exchange networks [25,26]. Finland has invested in workforce professional programmes as well as training offers during studies for doctors and nurses. A well-designed infrastructure supports the continual improvement of Finland’s *data sharing/IT* systems for VPC. For instance, public and private providers are required to report patient data to national data repositories [27]. Patient involvement is included in the digital strategy. Routine data collection surveys patients and providers [14]. Recent initiatives encourage patient involvement in their care as seen in primary care (Omalo platform) and secondary care via “Virtual Hospital”). (S1 Table).

#### Germany

In other federal countries such as Germany, their policy *objectives have* streamlined regulations at the national level [15]. This has had an impact on *regulation*, *where* a favourable legislative framework came into force in 2019 which contributed to improving the availability of remote consultations [28]. The country has aligned *financing and reimbursement* on par with face-to-face consultations. With regards to *delivery and integration*, the uptake of virtual care mostly happened among doctors already employed by the statutory health insurance system, facilitating the integration of face-to-face and digital care. There the development of private telemedicine companies moved at a slower pace due to the absence of provisions for them to bill the public health insurance system [29]. Doctors were explicitly forbidden to give medical advice solely based on virtual consultations until 2011 [15]. In Germany, patient involvement is mostly present under the form of empowering patients to better understand and have agency over their care. In the *workforce*, currently, video consultations are offered by self-employed doctors working under the statutory health insurance system mostly via teleconsultations platforms such as Doctolib. GPs dominate VPC system. There is no evidence of workforce training support in Germany. In Germany, there has been a concerted effort in the development of the electronic health card and patient electronic health records to support *data sharing/IT systems* [30]. The Nationwide eGK platform allows physicians to check patients’ data, upload and exchange reports and test results, thus facilitating data sharing; and since early 2021, the platform also gives patients access to their electronic health record (S2 Table). At the same time, concern for data protection and privacy led to a broad range of national level policies and initiatives to strengthen digitalisation [22,31,32]. Statutory health insurance primary care doctors tend to host video consultations on privately-owned telehealth platforms such as Doctolib which have their own embedded patient (and health professional) feedback forms and data collection methods.

#### Sweden

In Sweden, the decentralised system and *objectives in* decision-making takes place largely at the County Council level which leads to different implementation trajectories and requirements [18]. With regards to *financing and* reimbursement, negotiations resulted in lower tariffs for virtual care costing a third of face-to-face consultations. There, virtual consultations are reimbursed as out-of-county visits because private digital providers are reimbursed by the corresponding region on a per-consultation basis and there are co-payments for physical consultations [33]. In Sweden, *delivery and* integration issues relate to chronic difficulties of timely access to primary care, which led to a high demand for virtual consultations [34]; the rapid establishment of private telemedicine companies contrasts with the lack of similar options in the public sector [35]. There, younger, wealthier patients in urban settings with minor health issues report a positive experience [35]. Private providers in Sweden offer feedback forms and channels for their remote consultations, but they may not be suitable for all patients and conditions. GPs dominate the *workforce* in the VPC system. Some in Sweden raised concerns around the lack of training and support with working remotely as well as increased isolation [13]. Public and private systems operate separately and the lack of a nationally unified documentation system common to public and private providers limits the integration of services. Thus, *data sharing* and integration of care is an ongoing challenge. Private telemedicine apps such as Kry and MinDoktor ask patients to quickly rate their consultations (S1 Table, S2 Table).

#### United Kingdom (England)

With regards to *objectives*, in the UK, the health system is decentralised but with the arrival of Covid-19, specific policies were put in place centrally to support regional decision-making (England, Scotland, Wales, and Northern Ireland) (S1 Table, S2 Table). The impact on *regulation* in England, was that streamlining processes and procurement rules along with rapid responses from technology suppliers facilitated the spread of remote consultations in the early months of the Covid-19 pandemic [36]. In England, *financing and reimbursement* of remote consultations is on par with face-to-face consultations [37]. With regards to *delivery and integration*, private providers seem to attract younger, healthier and more affluent patients [38]. Most patients seem to report positive improvements with remote consultations, but in England older people and those with long-term conditions reported being less comfortable attending remote consultations [39]. GPs dominate the *workforce in* VPC delivery in the public health system while private providers for example in England offer the option of a consultation with a nurse. England established senior leadership positions of clinical information officers but there is insufficient investment in training during studies for data scientists and clinical informaticists [40]. GPs reported they enjoyed a better work life balance working for private providers who offer remote consultations. But the IT infrastructure is unevenly developed, measures to facilitate *data sharing* are lacking, and there is non-standardised collection and reporting of remote consultation data [41]. Patient involvement involves routine collection from the national GP survey which distinguishes whether the consultation was remote or in person. (S1 Table).

### Conceptual Framework for VPC

**Fig 1:**
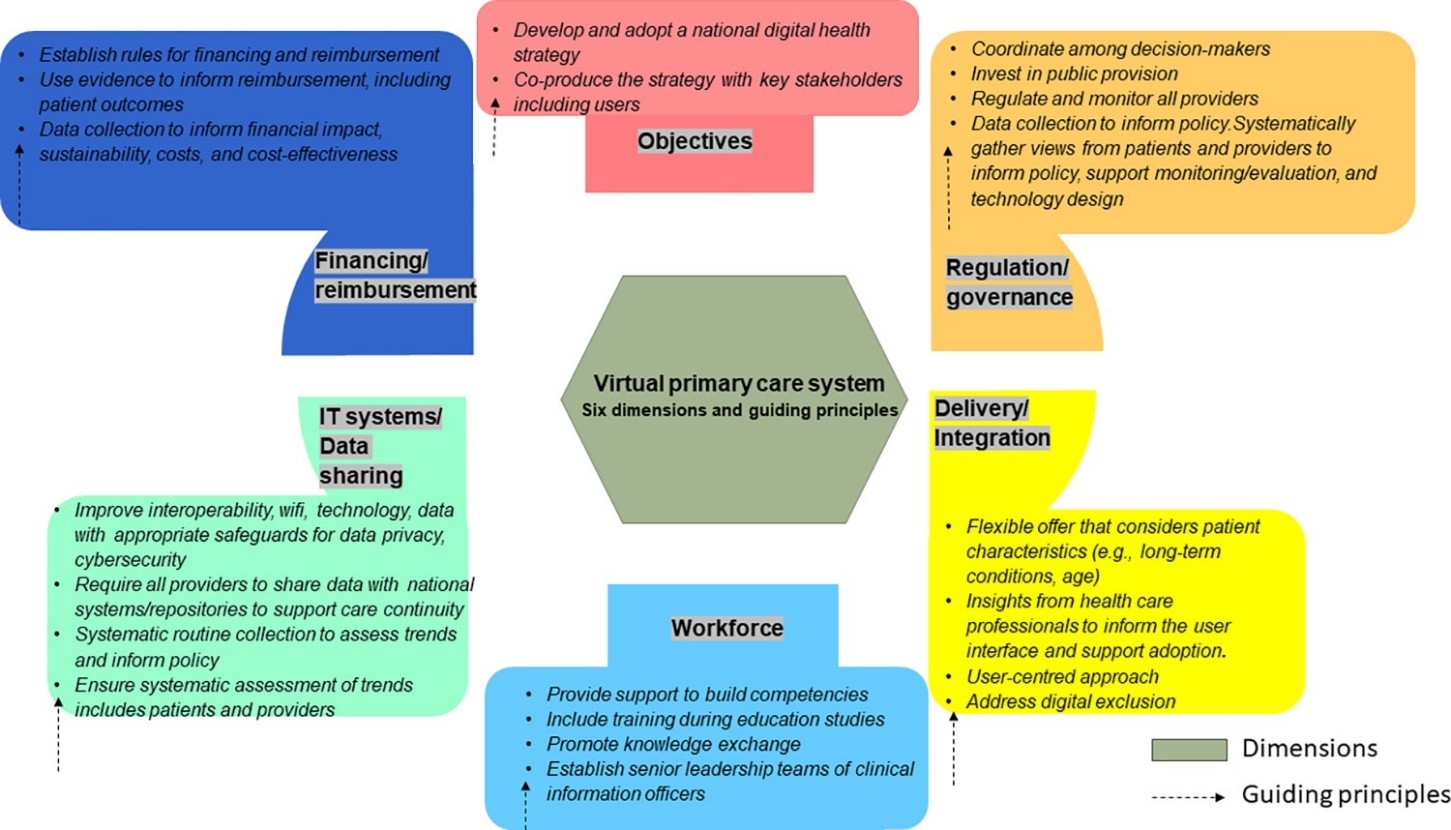
Digital health policy framework with guiding principles for a VPC system.

Each dimension of the framework sets out guiding principles to support a VPC system both in terms of its design but also its implementation (**Fig *1***) (S3 Table). The *Objective* dimension covers the need to develop a national digital strategy co-produced with key stakeholders to initiate the development and implementation of VPC. The *Regulation and governance* dimension captures the building of support structures to regulate and monitor providers and systematically gather user experience. The *Reimbursement and financing* dimension considers ways to support financing and reimbursement of VPC consultations. The dimension *Delivery and integration* covers the flexibility in the policy scope of the VPC system to meet user needs and mitigating digital exclusion. The *Workforce* dimension covers training during studies, support in the workplace such as upskilling, and team compositions (e.g., clinical information officers, and clinical informaticists). Finally, the dimension *IT systems and data sharing* covers interoperability with access to Wi-Fi technology and data with appropriate safeguards in place for data sharing to support continuity of care with routine data collection (e.g., user uptake, experience). This issue is of particular importance to support and address disparities and inequalities of access with regards to reliable internet and digital literacy for underserved populations.

The conceptual framework is accompanied by a country profile template to support evidence gathering with accompanying sub-research questions (Table 3). The template included the following aspects: policy objectives, regulatory landscape, financing and reimbursement policies, type of VPC system, its mode of delivery, workforce training requirements, data sharing and IT, and patient involvement.

**Table 3:** County profile template of VPC arrangements.

Framework domain	Sub-questions
Objectives and policy context	• What are the country’s policy objectives to deliver VPC (e.g., speed, experience, health literacy, accessibility, diversity, health inequalities, workforce needs/skill mix)? • What was the catalyst for these policies?
Regulation	• Are there institutions with a remit on VPC regulation? • What are the regulatory arrangements/requirements between VPC public/private provision, and for in-person/VPC consultations?
VPC system	• What kind of VPC services are being used in primary care settings (e.g., telemedicine, communication tools, AI tools, text)? • What platforms and software are used to enable patient-doctor communication? • How is patient involvement encouraged?
Delivery	• How do in-person and VPC services interact with each other? • Are VPC services integrated (i.e., practitioners offer both modes of consultation, patient data is shared between physical and virtual providers) or do VPC services run in parallel to physical services as a form of competitor?
Workforce	• What are the staffing arrangements (e.g., virtual vs in-person)? • Do physicians typically share their time between virtual and physical appointments or not? • Are workforce needs considered (e.g., coaches, skill mix, therapists)?
Financing/Reimbursement	• What are the reimbursement arrangements for in-person and VPC? • Is the reimbursement level similar for public and private providers who offer VPC consultations?
IT systems/data	• Are there parallel patient records for VPC public/private provision? • Does data sharing occur or between VPC public/private provision?

The conceptual framework that was derived from these findings offers a set of guiding principles that can facilitate the assessment of VPC in health system settings. Our framework is intended for decision-makers, to inform governments, digital health developers and its users. Both the framework and guiding principles offer a critical approach to assess country features and policy developments in VPC.

All five countries have taken different approaches with the deployment of VPC (Table 4). Country policy strategies discuss the need for improved leadership and governance, strong regulation and monitoring processes to ensure standards are met, investment in IT systems, training the healthcare workforce to improve the digital literacy of staff, as well as ensuring accessibility and portability of patient information across healthcare settings while adhering to privacy safeguards with data sharing [17–22].

**Table 4:** Summary of key features of VPC systems and opportunities in the VPC systems.

Country	Public or private delivery	VPC features	Delivery and integration	Workforce needs/skill mix offered	Improve Data sharing/IT across public and private	Regulation and governance	Pricing and reimbursement evaluations
**Canada**	Public/private mix	Depends on the province. Examples include: • Platforms used by public providers across the country: Microsoft Teams, Zoom • Private providers across the country: Maple, Babylon, Thrive • In several provinces: BASE e-consult platform (asynchronous consultations) • Ontario Telemedicine Network (OTN) in Ontario (public provider)	Need user feedback on a systematic basis	Absent	Needed	Streamline requirements Monitoring and evaluation of private and public platform	Absent
**Finland**	Public more widespread than private	• Public remote consultation widespread since the pandemic and monitored since 2013 • Private provision for occupational health private health care: Doctor online (Terveystalo)	Requires sub-group feedback by distinction in data collections of the type of technology used in VPC system	Absent	NA	NA	Absent
**Germany**	Private providers used in the public health system	• Over 25,000 practices in Germany offer virtual consultation Physicians must use one of over 30 certified video service providers (which include *Doctolib*, *Kry*) and have to notify their association. GPs account for 70% of video consultation users on *Doctolib*,	Integrate video consultations into the national Gematik platform to improve coordination of care for patients	Absent	Needed	Streamline requirements Monitoring and evaluation of private and public platform	Absent
**Sweden**	Private providers in public and private facilities	• Main private providers delivered in public and private facilities: Kry, Min Doktor, Doktor.se	Need user feedback and centred approach	Absent	Needed	Streamline requirements Monitoring and evaluation of private and public platform	Absent
**England**	Private providers in public/private facilities	• Public remote consultation widespread in GP practices (99% coverage) since the pandemic • Common platforms: Attend Anywhere, Accrux, NHS Near Me and MS Teams) Private providers contract with the NHS system: *Babylon GP at Hand*, *Livi*, *Pushdoctor* and via OOP or private health insurance) (*Livi*, *Pushdoctor)*	Improve user feedback on a systematic basis	Not at scale	Needed	Coordination, communication hinders clear national steer	Absent

## Discussion

This article aimed to advance the WHO building blocks framework to be better fit for purpose in the context of digital transformations in health, in particular VPC. Various implementation issues were identified through the updated framework and narrative review for all five countries. First, for-profit telemedicine has been difficult to regulate in some of the countries reviewed, with new arrangements (often temporary) having to be found with the private sector. Second, difficulties with regards to the coordination and continuation of care remain, particularly due to the lack of integration and data sharing between the public and private VPC systems. Third, disparities and inequalities of access remain a key issue, especially with regards to access to reliable internet and digital literacy [42], [43], particularly for older patients, patients with complex conditions, patients living in rural areas, and lower-income individuals. Fourth, current policies are inadequate to address issues like isolation among GPs who provide remote consultation, the need to develop eHealth competencies in education curricula and ongoing workforce training and support. Fifth, reimbursement has not been sufficiently addressed: fee-for-service is not optimal but remains a widely used payment method for telemedicine. Alternative methods of payment exist but there is less evidence available of how well they work in practice. Finally, reports of inadequate and insufficient infrastructure and lengthy bureaucratic and procurement processes hindered rapid roll-out. These findings align with previous research on digital health in other parts of the healthcare sector [44,45], indicating that these barriers are not specific to the field of primary care and instead may need to be addressed through a more holistic health policy lens [46]. There are opportunities for each country to improve the current use of VPC within the national health system. For example, learning from the large volumes of data being generated both at the national and international level on real-world data and real-world evidence [47].

### Country findings

#### Canada

In Canada, there is a need for greater focus on streamlining requirements for VPC systems across the country, strengthening interoperability, putting a greater emphasis on user-centred approaches, and increasing support for health professionals in their training [8,20]. The provincial remit in health presents a challenge in differing approaches to implementation but also an opportunity to learn from varying practices [8]. Strong monitoring and evaluation plans are needed to help inform financial and reimbursement policies. User feedback is used by the private sector to continuously shape digital services, but patients are insufficiently involved in co-creating digital solutions.

#### Finland

In Finland, the government has prioritised the digital health infrastructure over several years [14,27]. For example, the prioritisation in the national repositories of data in primary care has laid the groundwork to understand the impact of VPC systems at an aggregate level. At the same time, a decentralised approach has led to varying degrees of uptake. In particular, further work is needed to support population sub-groups in accessing the VPC system to mitigate inequities in access. For example, on the type of technology used in the VPC and differential impact among population sub-groups.

#### Germany

In Germany, the federal push towards remote consultations has contributed to its widespread availability. For example, over 25,000 practices offer a virtual consultation; but there is a need for more training and support of healthcare professionals in using these platforms [15]. Integrating video consultations with the national Gematik platform (which hosts patient electronic health records) would further facilitate coordination of care for patients and improve interoperability and data sharing where appropriate. The monitoring and evaluation of private and public platforms would support regulation of VPC systems and inform financing and reimbursement policies.

#### Sweden

In Sweden, further policy work is needed to ensure better coordination of care by integrating services, including coordinating IT systems and improving data sharing for better regulatory oversight of the private platform providers of VPC systems, as well as evaluating the current modes of payment and reimbursement of virtual consultations [18]. Building and fostering cooperation between providers, and increasing learning and development opportunities with proper peer support for health care workers is needed. A focus in improving inclusion and strengthening a user-centred approach would inform the monitoring of VPC system but also identify areas to improve access to population sub-groups.

#### United Kingdom (England)

In England, the multiplicity of decision-making bodies poses a real challenge with coordination and communication, making it difficult to provide a clear national steer to facilitate the implementation of a robust VPC system [36]. For example, a more focussed approach to collect information on the use of VPC system in both private and public settings including user feedback would inform the regulatory response and inform pricing and reimbursement policies. There is a need for greater policy focus to address the bureaucratic, procurement processes, inadequate infrastructure and insufficient workforce training and support.

### Limitations

There are limitations in our analysis that should be noted. Our study focussed on five countries to capture differences in the policy development, implementation and delivery of VPC systems. The narrative review was not systematic in design. The analysis is based on published available information and did not capture policy changes beyond 2022. We are unable to verify the published information with corroboration from field experts, though we did draw on expertise of the study team to compliment the narrative review. We note that the referenced publications are predominantly in English and so is not exhaustive of publications in each country’s native language, which may be especially relevant for our collection of grey literature. This framework has implications to understand secondary and tertiary care services, particularly around coordination of care involving electronic health records for example and is an important policy area worthy of future work but was outside the scope of this analysis [44].

## Conclusion

In conclusion, the implementation of VPC within a health system is a complex challenge that is contingent on many stakeholders. Our proposed digital health policy framework and guiding principles can be a useful starting point to assess how VPC systems are working in practice. The growing interest in VPC suggests that decision-makers should consider a flexible VPC offer; one that is more appropriate at identifying patients and draws on user experience to inform the design of its delivery, the health impacts and particularly for underserved populations including affordability of digital devices, and connectivity. A policy to expand VPC policies at scale requires consideration of the financial sustainability including pricing and reimbursement of VPC, the cost of putting in place the required infrastructure, data needs and a well-trained workforce to support its delivery. Ultimately, a well-designed primary care system forms the cornerstone of an effective health system. In the face of rising healthcare costs and health workforce shortages worldwide, it is vital that VPC can be mobilised effectively without the risk of exacerbating existing inequalities or further skyrocketing healthcare costs.

## Supporting information

S1 Table Delivery/integration and workforce features of VPC systems. (PDF)

S2 Table Regulation/governance, financing and reimbursement and data sharing/IT systems of VPC systems. (DOCX)

S3 Table Proposed digital health policy framework with guiding principles for a VPC system. (PDF)
