# Supplementary material for: A Framework for Digital Health Policy: Insights from Virtual Primary Care Systems Across Five Nations
Source: PLOS Digit Health. 2023 Nov 8;2(11):e0000382. doi: 10.1371/journal.pdig.0000382 (PMC10631700; doi:10.1371/journal.pdig.0000382)
Supplement: S1 Table — (PDF) [file pdig.0000382.s001.pdf]

S1 Table. Delivery/integration and workforce features of VPC systems

Country	Accessibility	Patient involvement	Health care professionals involved in delivery	Workforce needs/skill mix
**Canada**	• Patients within the statutory health insurance system access virtual care either via their practice if it offers it, or via another one that does within the same province if theirs does not, although on a more ad-hoc/temporary basis.<br>• Patients covered by private insurance, mostly through company plans, are covered for virtual consultations via certain platforms/apps.	• Patient feedback on VPC consultations was absent in most cases due to the use of phone calls rather than apps or video consultation platforms by traditional GPs.	• GPs and nurses<br>• Physicians are restricted to practice telemedicine in the province or territory in which they hold a license.<br>• Physicians delivering care through private telemedicine apps such as Akira are generally employed on contract, part-time, directly by the company.	• Little literature around the experiences and needs of Canadian healthcare professionals in VPC.<br>• No information on the training provided; training is absent in provincial digital health strategies.
**Finland**	• Uptake and use vary across age and gender but increased across all ages since the pandemic<br>• Underserved populations a challenge	• Patient involvement included in the digital strategy supported with a well-built infrastructure<br>• Design of surveys involve patient groups as a key stakeholder<br>• Systematic and routine data collection gather patient feedback and experience	• GPs and nurses<br>• Nurses had the highest remote contacts in public municipal health centres<br>• GPs and nurses are employed in public municipal health centres<br>• GPs can have both public and private practice	• Investment in workforce with professional programmes for ehealth competency for doctors, dentists and nurses.<br>• University led work in medical programmes (ehealth training package)

		• Systematic analysis of data and trends are used to inform policy<br>• Recent initiatives encourage patient involvement in their care as seen in primary care (Omalo platform) and secondary care via "Virtual Hospital")	• Older GPs with experience are employed by private providers	
**Germany**	• Available to all individuals covered by the public health insurance system.<br>• Widespread support for remote consultations to continue after the pandemic	• Effort towards increasing patient engagement with their own care via access to online electronic health records (ePA) as well as access to their health account via the eGK app.<br>• Statutory health insurance primary care doctors tend to host video consultations on privately-owned telehealth platforms such as *Doctolib* which have their own embedded patient (and health professional) feedback forms and data collection methods.	• Mostly GPs<br>• Almost exclusively self-employed, statutory health insurance physicians, delivered to their usual patients.	• Not all German medical faculties offer courses on digital health and digital competencies. Existing courses vary in scope and are frequently part of the elective rather than the compulsory curriculum.<br>• No literature available around the experiences of and training opportunities for primary care doctors delivering virtual care.

**Sweden**	•Mostly used by younger, wealthier people in urban settings with minor health issues with positive experience<br>•Underserved populations a challenge	•Private telemedicine apps such as Kry and MinDoktor ask patients to quickly rate their consultations.	• Mostly GPs<br>• Triage by nurses, other health care professional growing<br>•Employed full-time, part-time or per hour and can combine working at a physical clinic (public or private)	•Lack of technical and professional support and social isolation have been reported in various studies of healthcare professionals' experiences.
**United Kingdom**	•Uptake and use increased during the pandemic but those with long-term conditions are less comfortable attending a remote consultation<br>•Underserved populations a challenge<br>• Some examples of collaborative delivery efforts with public, private and voluntary sector underway to improve digital inclusion and digital literacy in England and Wales	• Patient involvement increased in the public system out of necessity due to the pandemic (Accrux, Second Nature, Oviva, Exi, Econsult).	• GPs	• Recent investment in workforce training and support for health care workers (NHS Digital Academy, establishing senior leadership positions clinical information officers<br>• Insufficient investment in training during studies for data scientists and clinical informaticists
