# Supplementary material for: A Framework for Digital Health Policy: Insights from Virtual Primary Care Systems Across Five Nations
Source: PLOS Digit Health. 2023 Nov 8;2(11):e0000382. doi: 10.1371/journal.pdig.0000382 (PMC10631700; doi:10.1371/journal.pdig.0000382)
Supplement: S2 Table — (DOCX) [file pdig.0000382.s002.docx]

**S2 Table. Regulation/governance, financing and reimbursement and data sharing/IT systems of VPC systems**

| Country | Regulation and governance | Financing and reimbursement | Data sharing and IT systems |
| --- | --- | --- | --- |
| **Canada** | - Federation of Medical Regulatory Authorities of Canada (FMRAC) develops legal requirements nationally, but guidelines such as which platforms to use are drafted by regional health authorities. | - During the pandemic, the introduction of temporary billing codes allowed doctors to bill provincial health plans for video and telephone consultations at the same rate as in-person visits. - Most private providers offer virtual consultations for CAD 49 as well as company subscriptions. | - Limited data sharing. - Same personal identifier (provincial health card) used by both public and private providers is the only form of data integration. |
| **Finland** | - MSAH provides overall steer working with other stakeholder: THL, Kela, Valvira for example to set out standards in the provision of remote services. | - Since 2016 reimbursement for physicians, physiotherapists, psychologists and nurses. - In 2020, private health care physicians accounted for 1.5% of all reimbursements. - OOP (Out of Pocket) with a private provider starts at EUR 36.10 for a chat with a doctor, EUR 54.10 video appointment, EUR 20 for chat with a nurse | - Public led investment in infrastructure in information technology over many years - Data flow to national data repositories (e.g., My Kanta, Avohilmo) allow for data sharing, tracking and monitoring trends to inform digital health strategy - Improved interoperability between regions - Work in progress between health and social care integration |
| **Germany** | - National/federal oversight, which helps streamlining processes and requirements across the country. - German regions have a strong degree of autonomy in health care system planning and provision | - Flat reimbursement rate via the use of billing codes, with an additional fee for the authentication of patients at the beginning of the online consultation. | - Approval process (which assesses the clinical, safety and financial aspects) for telehealth platforms (and other e-health projects) before inclusion in the Vesta directory for interoperability. - Nationwide eGK platform allows physicians to check patients’ data, upload and exchange reports and test results, thus facilitating data sharing. - Since early 2021, the platform also gives patients access to their electronic health record. |
| **Sweden** | - Swedish public healthcare system operates at regional (county) level. - The country’s 2009 freedom-of-choice law facilitated entry of private companies that operate nationally | - Reimbursed as out-of-county visits since 2017, cost the system only 1/3^rd^ of the price of physical consultations (SEK 650 – EUR 65) with a similar copayment (SEK 250 – EUR 25). - For OOP for private providers, similar co-payment as for F2F consultations (SEK 250 – EUR 25). | - Integration in the public system is facilitated by the use of national ID numbers - Limited data sharing due to a lack of a unified documentation system common to both public and private providers - Private telemedicine companies are not allowed to enlist patients |
| **United Kingdom** | - Health care responsibility devolved to the four nations’ health authorities. - Some cross-over working with UK bodies such as the MHRA, ICO | - GP practices receive a risk adjusted capitated payment for all patients registered at their practice (e.g., approx. GBP 151 in England) with no distinction or additional incentives for virtual consultations - Examples of OOP with a private provider are GBP 29 (*Livi), and* GBP 45 (*Pushdoctor)* | - Uneven spread of well-developed IT infrastructure, lack of measures to facilitate linkage between datasets, and non-standardised collection and reporting of remote consultations are persistent challenges |
