# Supplementary material for: A Framework for Digital Health Policy: Insights from Virtual Primary Care Systems Across Five Nations
Source: PLOS Digit Health. 2023 Nov 8;2(11):e0000382. doi: 10.1371/journal.pdig.0000382 (PMC10631700; doi:10.1371/journal.pdig.0000382)
Supplement: S3 Table — (PDF) [file pdig.0000382.s003.pdf]

S3 Table. Proposed digital health policy framework with guiding principles for a VPC system

Proposed digital health policy framework with guiding principles for a VPC system	
**Policy objectives**<br>• Develop and adopt a national digital health strategy<br>• Co-produce the digital health strategy with key stakeholders (e.g., relevant government authorities, in health policy and planning, data collection and analysis, health professionals, patient groups)	**Regulation and governance**<br>• Support the public provision of VPC systems.<br>• Regulate and monitor providers (including those in the private sector).<br>• Draw on systematic data collection to inform policy. Gather views from patients and providers to inform policy, support monitoring, evaluation and technology design.<br>• Ensure coordination and communication among decision-makers.
**Financing and reimbursement**<br>• Establish rules for financing and reimbursement for VPC. Draw on evidence including patient outcomes.<br>• Systematic financial data collection on remote consultations to gather evidence on financial impact, costs, sustainability and cost effectiveness to inform policy.	**Delivery and integration**<br>• Promote a flexible offer that considers when VPC is appropriate for patient groups.<br>• Gather insights from health care professionals to inform the user interface and support adoption.<br>• Adopt a user-centered approach for both patients and health care professionals.<br>• Address digital exclusion.
**Workforce training, support and planning**<br>• Include virtual care training during health care professional educational studies.<br>• Provide workplace technical and professional support for digital upskilling.<br>• Promote knowledge exchange networks.<br>• Team composition mix that is inclusive e.g., clinical information officers in leadership teams, clinical informaticists, data scientists.	**IT systems and data sharing**<br>• Improve interoperability, access to Wi-Fi, technology, and data with appropriate safeguards in place for data sharing.<br>• Require all providers (including private) to share patient data with national systems and data repositories to support continuity of care.<br>• Embed routine data collection to assess trends inform policy. Ensure the systematic assessment of trends includes patients and providers (e.g., uptake, participation, experience).
